# The mediating role of self-efficacy in the relationship between Big five personality and depressive symptoms among Chinese unemployed population: a cross-sectional study

**DOI:** 10.1186/1471-244X-14-61

**Published:** 2014-03-03

**Authors:** Yang Wang, Lutian Yao, Li Liu, Xiaoshi Yang, Hui Wu, Jiana Wang, Lie Wang

**Affiliations:** 1 Department of Social Medicine, School of Public Health, China Medical University, No. 92 Beier Road, Heping District, Shenyang, Liaoning 110001, People’s Republic of China

**Keywords:** Depressive symptoms, Big five personality, Self-efficacy, Unemployed

## Abstract

**Background:**

Besides the rapid growth of economy, unemployment becomes a severe socio-economic problem in China. The huge population base in China makes the unemployed population a tremendously huge number. However, health status of unemployed population was ignored and few studies were conducted to describe the depressive symptoms of unemployed individuals in China. This study aims to examine the relationship between Big five personality and depressive symptoms and the mediating role of self-efficacy in this relationship.

**Methods:**

This cross-sectional study was performed during the period of July to September 2011. Questionnaires consisting of the Center for Epidemiologic Studies Depression Scale (CES-D), the Big Five Inventory (BFI) and the General Self-efficacy Scale (GSE), as well as demographic factors, were used to collect information of unemployed population. A total of 1,832 individuals (effective response rate: 73.28%) became our subjects. Hierarchical linear regression analyses were performed to explore the mediating role of self-efficacy.

**Results:**

The prevalence of depressive symptoms was 67.7% among Chinese unemployed individuals. After adjusting for demographic characteristics, extraversion, agreeableness and conscientiousness were all negatively associated with depressive symptoms whereas neuroticism was positively associated with depressive symptoms. The proportion of mediating effect of self-efficacy in the relationship between extraversion/agreeableness/conscientiousness/neuroticism and depressive symptoms was 25.42%, 10.91%, 32.21% and 36.44%, respectively. Self-efficacy is a mediator in the relationship between extraversion/agreeableness/conscientiousness/neuroticism and depressive symptoms.

**Conclusion:**

Self-efficacy partially mediated the relationship between Big five personality and depressive symptoms among Chinese unemployed individuals. Interventions that focus on both individuals’ personality and self-efficacy may be most successful to reduce depressive symptoms of unemployed individuals.

## Background

Besides the rapid growth of economy, unemployment becomes a severe socio-economic problem in China. Giles et al. estimated that unemployment rate of urban permanent residents increased from 6.1% to 11.1% from January 1996 to September 2002 in China [1]. The huge population base in China makes the unemployed population a tremendously huge number. However, few studies were conducted to describe the health status of unemployed population in China.

Previous researches have indicated that the loss of employment was one of the factors that has considerable influence on health status [2-4]. Unemployment was found to be associated with alcohol consumption, somatisation, suicidal ideation and symptoms of physical illness [5] and resulted in the destruction of self-efficacy, which have harmful effects on unemployed’ health [6]. Studies have also demonstrated a positive correlation between employment and improved self-confidence, self-esteem, and happiness and people in secure employment [7] are reported to recover more quickly from illness than unemployed [8].

The relationship between unemployment and mental health has also been reported by a number of researches [2,4,9]. Unemployed people are very likely to experience psychological tension, mainly depression and anxiety, which negatively affects their health, their family’s security, and society’s stability in general [10]. Depression is more elevated among the long-term unemployed [11]. Krysia has indicated that longer durations of unemployment predicted higher levels of depressive symptoms among young adults in the U.S. [12].

Previous studies have indicated that personality was associated with depressive symptoms [13-15]. The Big five model is the most generally used dimensional model of personality. It includes five dimensions: extraversion (vs. introversion), agreeableness (vs. antagonism), conscientiousness (vs. lack of direction), neuroticism (vs. emotional stability) and openness (vs. closeness to experience). Extraversion was shown to be negatively associated with depression while neuroticism to be positively associated with depression [13]. Kotov et al. conducted a quantitative review to examine the associations between Big five personality and depression and found that depression groups score low on conscientiousness [14]. Bienvenu et al. found a positively association between openness of experience and depression [15]. However, little is known about these associations among unemployed population in China.

“Self-efficacy is the belief in one’s capabilities to organize and execute the courses of action required to produce given attainments” [16]. Since self-efficacy was introduced by Bandura, studies examining the relationship between self-efficacy and depression have showed that those who scored higher on self-efficacy are less likely to develop depressive symptoms [17,18]. Self-efficacy is not a static characteristics, as elaborated by Bandura. Theoretically, internal personal factors and external environment both can alter one’s self-efficacy. Self-efficacy is also reported as a positive resource for combating diseases such as diabetes [19], oncology [20] and osteoarthritis [21].

Although both the association between Big five personality and depressive symptoms and the association between self-efficacy and depressive symptoms have been confirmed by previous studies, the role of self-efficacy as a mediator between Big five and depressive symptoms has not been examined to our knowledge. A well-known theory in the field of stress is transactional theory proposed by Lazarus and Folkman in 1984 [22]. According to this theory, an individual’s reaction to the stress is mediated by the subjective evaluation (i.e., appraisal) of the environment and the process of coping with a stressful event. Appraisals of situations and coping behavior are influenced by personal characteristics such as personality, perceived control, self-efficacy, and social skills. Based on this theory, we considered not only the possibility that Big five personality predicts depressive symptoms, but also the possibility that the effect of Big five personality on the depressive symptoms is mediated through the effect of Big five personality on self-efficacy.

With the purpose of relieving the adverse effects of unemployment and enhancing unemployed’ health status, we conducted a cross-sectional survey among unemployed population to explore the relation of Big five personality and self-efficacy to depressive symptoms. There are three specific aims in the present study. First, to investigate the prevalence of depressive symptoms among unemployed individuals in China. Second, to examine the relation of Big five personality and self-efficacy to depressive symptoms among unemployed population in China. Third, to explore the mediating role of self-efficacy in the relationship between Big five personality and depressive symptoms among unemployed population in China.

## Method

### Procedure and sampling

A cross-sectional study was conducted in Mainland, China, from January to September 2011. Based on the geographic and administrative divisions of Mainland, China, all provinces and municipalities are divided into three regions: East regions, Central regions and West regions. Four provinces in each region were randomly selected (in Central regions, three provinces, along with the municipality of Beijing were selected). Two cities were randomly selected from each province. A total of 22 cities and 1 municipality were selected. Then, in each sampled city or municipality, unemployed people, aged 18 to 65 years old, who are jobless and have been actively looking for work within the past four weeks and registered in the labor markets of the local Human resources and Social Security Bureau, were enrolled in this study by convenience sampling in the local large labor markets (at least 300 Unemployed individuals were registered). Eventually, 2,500 unemployed participants were recruited in this study and a pool of 1,832 unemployed participants (effective response rate: 73.28%) constituted the potential study sample. After obtaining written informed consent to conduct this survey, the unemployed participants were interviewed face-to-face by trained interviewers from China Medical University. This study received ethics approval from the Committee on Human Experimentation of China Medical University.

Interviewers were enrolled voluntarily from the 4th year undergraduate students of China Medical University. A 2-day interviewer training was conducted by experts from School of Public Health, China Medical University. Sources of survey error, the role of interviewer in reducing error, interview techniques including standardized question asking, questionnaire format and conventions, clarification, feedback, answer recording, etc. were introduced to interviewers.

### Demographic characteristics

Gender, age, education, martial status, chronic disease, household monthly income and duration of unemployment were obtained in this study. “Education” was categorized as “middle school or under”, “senior high school” and “college or above”. “Marital status” was categorized as “single”, “married/cohabitation” and “divorced/separated/widow”. “Chronic disease” was defined as ‘yes’ if any of these chronic disease (e.g., hypertension, cardiovascular disease, diabetes, ulcer, chronic kidney disease, back pain and arthritis) had ever been diagnosed. Household monthly income was categorized as “≤1000rmb (≈158 dollars)”, “1001~2000rmb” and “>2000rmb). Duration of unemployment was categorized as “1-2 months”, “3-5 months”, “6-11 months” and “≥12 months”.

### Measurement of depressive symptoms

Depressive symptoms were measured by the Center for Epidemiologic Studies Depression Scale (CES-D) [23]. It consisted of 20 items and each item was scored on a 4-point Likert scale on the basis of frequency of occurrences of current depressive symptoms during the past week. Each item had four response categories ranging from ‘rarely or none of the time’ (0 points) to ‘most or all of the times’ (3 points), with the total score ranging from 0 to 60. Higher score indicated higher level of depressive symptoms. Subjects scored of 16 or more were defined as “depressive symptoms” group [24].

The Chinese version of the CES-D has been used widely in Chinese population and demonstrated satisfactory reliability and validity [25,26]. In the present study, the Cronbach’s alpha for the total scale was 0.884.

### Measurement of big five personality

Big five personality was measured by The Big Five Inventory (BFI), which was constructed by John, Donahue and Kentlein in 1991 [27,28]. It measured an individual on the Big Five Factors (dimensions) of personality which included extraversion (vs. introversion), agreeableness (vs. antagonism), conscientiousness (vs. lack of direction), neuroticism (vs. emotional stability) and openness (vs. closeness to experience). The inventory consisted of 44 items, and the extraversion dimension was measured by 8 items, the agreeableness dimension was measured by 9 items, the conscientiousness was measured by 9 items, the neuroticism dimension was measured by 8 items and the openness dimension was measured by 10 items. Each of the items is scored on a Likert scale from 1(strongly disagree) to 5 (strongly agree).

The Chinese version of the BFI has been used in Chinese population and demonstrated good reliability and validity [29,30]. In the present study, the Cronbach’s alpha coefficients of extraversion, agreeableness, conscientiousness, neuroticism and openness were 0.723, 0.759, 0.786, 0.753 and 0.714, respectively.

### Measurement of self-efficacy

Self-efficacy was measured by General Self-efficacy Scale (GSE), developed by Schwarzer [31]. The Chinese version of this scale showed good reliability and validity when used in Chinese population [32,33]. The scale consisted of 10 items and each item was scored on a 4-point Likert scale from 1(strongly disagree) to 4(strongly agree). Responses for all items were summed up to get an overall score and higher scores indicated higher level of self-efficacy. In the present study, the Cronbach’s alpha for the total scale was 0.756.

### Statistical analysis

All analyses were conducted using SPSS 17.0 for Windows. Pearson’s correlation coefficient was used to preliminarily examine the correlations among depressive symptoms, big five and self-efficacy.

Hierarchical multiple regression (HMR) analysis was performed to test the incremental variance of any given set of independent variables and to examine the mediating role of self-efficacy. Scores of depressive symptoms were used as dependent variables. The independent variables were entered in three steps. In step 1, all demographic variables were entered as control variables. Because marital status and educational level are categorical variables without a linear trend, we set dummy variables for the two variables respectively. For marital status, “married” was set as the reference group. For educational level, “middle school or under” was set as the reference group; in step 2, dimensions of Big five personality were added; in step 3, self-efficacy was added. The analysis was performed in stages by successively inputting several blocks of independent variables in the model. All statistical tests were two-sided (α = 0.05).

Baron and Kenny’s analysis technique [34] was used for testing the hypothesis concerning the mediating effect of self-efficacy in the relationship between the dimensions of big five personality and depressive symptoms. According to Baron and Kenny, the following are the conditions for establishing mediation: (1) the independent variable (Big five personality) is significantly related with the dependent variable (depressive symptoms); (2) the independent variable (Big five personality) is significantly related with the mediator (self-efficacy); and (3) the mediator (self-efficacy) is significantly related with the dependent variable (depressive symptoms), with the effect of the independent variable (Big five personality) on the dependent variable (depressive symptoms) shrinking (partial mediator) or becoming statistically insignificant (full mediator) upon the addition of the mediator (self-efficacy) to the model.

All the continuous variables were standardized in order to avoid multicollinearity [35] before performing the regression analyses.

In addition, bootstrapping method was performed to confirm the mediation effect. Bootstrapping is an increasingly popular non-parametric method of testing mediation effect [36]. It involves repeatedly sampling from the data set and estimates the indirect effect in each resampled data set. It provides a powerful and reasonable method of obtaining confidence interval (CI) for mediation effect under most conditions. For each independent variable, when the bias-corrected and accelerated 95% CI (BCa 95% CI) of medication effect (a*b product) excluded 0 , it indicated that the mediating role of self-efficacy was statistically significant. To estimate the degree to which the effect of Big five personality on depressive symptoms is mediated through self-efficacy, we also calculated the proportion of the indirect effect of Big five personality on depressive symptoms to the total effect. The bootstrap estimate presented in our study was based upon 5,000 bootstrap samples.

## Results

### Demographic characteristics of studied population and prevalence of depressive symptoms

Demographic characteristics of the subjects and the distributions of depressive symptoms in categorical variables are shown in Table 1. The mean age of studied population was 34.88 years old (SD = 10.59) and 56.1% of studied population were males. The mean score of depressive symptoms among the unemployed population in the present study was 21.39 and the prevalence of depressive symptoms was 67.7%.

**Table 1:** Demographic characteristics of study subjects and the distributions of depressive symptoms in categorical variables

**Variable**	**N(%)**	**Depressive symptoms**
		**Mean (SD)**
Gender		
Males	1027(56.1%)	21.62(10.81)
Females	805(43.9%)	21.10(10.23)
Age (yr)		
≤30	778(42.5%)	20.30(10.04)
31-40	458(25.0%)	22.21(10.57)
>40	596(32.5%)	22.19(11.08)
Education		
Middle school or under	597(32.6%)	23.69(11.68)
Senior high school	567(30.9%)	21.03(10.06)
College or above	668(36.5%)	20.10(9.67)
Marital status		
Single	593(32.4%)	20.01(9.87)
Married/cohabitation	1076(58.7%)	21.58(10.91)
Divorced/ separated / widow	163(8.9%)	25.20(9.58)
Chronic disease		
Yes	834(45.5%)	23.27(10.34)
No	998(54.5%)	19.85(10.51)
Household monthly income		
≤1000rmb	421(23.0%)	24.80(11.76)
1001~2000rmb	815(44.5%)	21.44(9.38)
>2000rmb	597(32.6%)	18.94(10.54)
Duration of unemployment		
1-2 months	386(20.1%)	18.33(9.86)
3-5 months	372(20.3%)	21.40(9.52)
6-11 months	454(24.8%)	22.47(10.01)
≥12 months	620(33.8%)	22.59(11.69)

### Correlations among Big five personality, self-efficacy and depressive symptoms

As seen in Table 2, extraversion, agreeableness, conscientiousness and openness were all negatively related with depressive symptoms whereas neuroticism was positively related with depressive symptoms. Therefore, the first condition of Baron and Kenny’s technique to test the mediating role of self-efficacy was satisfied in the present study. Results in Table 2 also showed that extraversion, agreeableness, conscientiousness and openness were all positively related with self-efficacy whereas neuroticism was positively related with it. Thus, the second condition of Baron and Kenny’s technique was also met.

**Table 2:** Means, standard deviations (SD) and correlations of continuous variables

**Variables**	**Mean**	**SD**	**1**	**2**	**3**	**4**	**5**	**6**
1. Depressive symptoms	21.39	10.56						
2. Extraversion	25.94	4.13	-0.291**					
3. Agreeableness	31.93	4.82	-0.415**	0.244**				
4. Conscientiousness	30.74	4.75	-0.349**	0.316**	0.516**			
5. Neuroticism	24.24	4.25	0.435**	-0.319**	-0.327**	-0.341*		
6. Openness	31.94	5.21	-0.211**	0.347**	0.370**	0.398**	-0.131**	
7. Self-efficacy	38.29	5.69	-0.494**	0.356**	0.377**	0.395**	-0.516**	0.335**

*P<0.05.

**P < 0.01 (two-tailed).

### Association between Big five personality and depressive symptoms

Results of the hierarchical multiple regression models of depressive symptoms were presented in Table 3. Each block of the independent variables made a significant contribution to the variance of depressive symptoms. Big five personality explained 24.7% of the variance in the depressive symptoms while demographic characteristics including gender, age, marital status, education, chronic disease, household monthly income and duration of unemployment contributed 8.7% of the variance. After controlling for demographic characteristics, extraversion (β = -0.111, P < 0.001), agreeableness (β = -0.219, P < 0.001), conscientiousness (β = -0.099, P < 0.001) were positively associated with depressive symptoms whereas neuroticism (β = 0.280, P < 0.001) was negatively associated with it.

**Table 3:** Hierarchical linear regression analysis results

**Variables**	**Depressive symptoms**		
	**step 1(β)**	**step 2(β)**	**step 3(β)**
**Block 1**			
Gender	-0.026*	-0.018	-0.026
Age	-0.077	0.009	0.007
Dummy_m1	-0.041	-0.040	-0.025
Dummy_m2	0.103**	0.046*	0.042*
Dummy_e1	-0.068*	-0.036	-0.026
Dummy_e2	-0.074*	-0.039	-0.029
Chronic Disease	0.113**	0.059**	0.061**
Household monthly income	-0.172**	-0.124**	-0.107**
Duration of unemployment	0.085**	0.063**	0.060**
**Block 2**			
Extraversion		-0.111**	-0.083**
Agreeableness		-0.219**	-0.195**
Conscientiousness		-0.099**	-0.067**
Neuroticism		0.280**	0.178**
Openness		0.020	0.060**
**Block 3**			
Self-efficacy			-0.264**
R^2^	0.087	0.334	0.377
Δ R^2^	0.087	0.247	0.043

*P < 0.05**P < 0.01 (two-tailed).

Dummy_m1 means “Single” vs. “Married/Cohabitation”, Dummy_m2 means “Divorced/Widow/Separated” vs. “Married/Cohabitation”;.

Dummy_e1 means “Senior high school” vs. “Middle school or under”, Dummy_e2 means “College or above” vs. “Middle school or under”.

### Association between self-efficacy and depressive symptom

The effect of self-efficacy on depressive symptoms was negative and significant (β = -0.264, P < 0.001), accounting for an additional 4.3% of the variance of depressive symptoms.

### The mediating role of self-efficacy in the relationship between Big five personality and depressive symptoms

After adding self-efficacy in the regression model, the regression coefficients (absolute value of regression coefficient when it is negative) for extraversion (from β = -0.111 to β = -0.083 , P < 0.001), agreeableness (from β = -0.219 to β = -0.195, P < 0.001), conscientiousness (from β = -0.099 to β = -0.067, P < 0.001) and neuroticism (from β = 0.178, P < 0.001) all diminished as shown in the final model. Based on the third condition of Baron and Kenny’s technique, we can see that self-efficacy is a partially mediator in the relationship between extraversion/agreeableness/conscientiousness/neuroticism and depressive symptoms.

Results of bootstrapping method showed that path coefficients of indirect effects (a*b product) of extraversion/agreeableness/conscientiousness/neuroticism on depressive symptoms through self-efficacy were -0.029 (BCa 95% CI: -0.044,-0.017), -0.024 (BCa 95% CI: -0.039,-0.011), -0.032 (BCa 95% CI: -0.049,-0.018), 0.103 (BCa 95% CI: 0.080,0.128), respectively. The proportion of indirect effect to total effect of extraversion/agreeableness/conscientiousness/neuroticism was 25.42%, 10.91%, 32.21% and 36.44%, respectively. The mediating effect of self-efficacy in the relationship between neuroticism and depressive symptoms was the strongest.

## Discussion

This study investigated the prevalence of depressive symptoms among Chinese unemployed population, explored the relationship between Big five personality and depressive symptoms and the mediating role of self-efficacy in this relationship. In this research, we found that 67.7% of Chinese unemployed individuals had depressive symptoms. This prevalence was almost two times higher than that was found in general populations in Japan [37] and Korea [38] and also higher than the prevalence for depressive disorders in the older, long-term unemployed population (38.5%) in German [39].

In the present study, results of the last model of HMR indicated that extraversion, agreeableness and conscientiousness were all negatively associated with depressive symptoms whereas neuroticism was positively associated with depressive symptoms among Chinese employed population, when adjusting for demographic characteristics and self-efficacy. The association between openness and depressive symptoms was initially non-significant, but became positively significant when self-efficacy was included in the model. Of the Big five personality, neuroticism and extraversion are the two major dispositions reflecting individual differences in the degree to which one experiences overall negative and positive affect, respectively. Individuals who score high on neuroticism are more self-conscious and more prone to feelings of inadequacy, worry and nervousness [14]. Compared to individuals with lower scores on neuroticism, they are more likely to experience negative emotions such as angry, anxiety, helplessness, fearfulness and depression [13]. McCann reported that neuroticism even accounted for 32.0% of the variance in suicide rates when demographics and depression were controlled [40]. The strong relationship between neuroticism levels and stress might due to a highly inability of individuals with higher scores on neuroticism to cope effectively with stressors, which would increase the amount of stress [13]. In contrast, extraversion seems to encompass sociability, enthusiasm, cheerfulness and reports of subjective wellbeing [41]. Highly extraverted individuals would suffer less under stress, as they would appraise stressful events as challenges and they see situations as opportunities for reward, not for punishment [39]. Researches have revealed that extraverted individuals reported less perceived stress and depression [42,43].

Results of the present study showed that self-efficacy played a positive role in alleviating depressive symptoms. This was in agreement with conclusions from previous studies that efforts to establish and maintain a sense of control over one’s life and environment might serve to modify the appraisal of stressful circumstances and to build a certain degree of resistance to depressive symptoms [17,44]. A strong sense of self-efficacy enhances personal accomplishments, reduces stress and lowers vulnerability to depression. In contrast, individuals with low self-efficacy doubt their capacities and have low aspirations and weak commitment to the goals they choose to pursue [45]. They are more vulnerable to stress and depression [16,17,46]. Self-efficacy development has been paid much attention among adolescences and patients [45,47], however, programs targeted toward developing unemployed population’ self-efficacy are few. To our knowledge, no self-efficacy interventions designed for unemployed have been conducted yet in China.

Big five personality not only had direct effect on depressive symptoms, but also had indirect effect on depressive symptoms through self-efficacy. In the present study, self-efficacy was found to partially mediate the relationship between extraversion/agreeableness/conscientiousness/neuroticism and depressive symptoms. Unemployed individuals who have higher scores on extraverted/agreeable/conscientious may have high self-efficacy, which in turn would lead to lower levels of depressive symptoms. And unemployed individuals who have higher scores on neuroticism may have low self-efficacy, which in turn would lead to higher levels of depressive symptoms. These findings suggested that interventions that focus on both individuals’ personality and self-efficacy may be most successful to reduce depressive symptoms of unemployed individuals. A shift in stress and health research now called for is from managing health risks to developing individuals’ strength and resilience. It is possible to modify responses of individuals who have higher scores on neuroticism through increasing awareness toward maladaptive ones and training coping self-efficacy. For example, cognitive-behavioral therapies and hardiness training have been shown to be effective in enhancing adaptive types of coping, perceptions of social support and attenuating psychological distress [48]. Administrators in different levels of Human resources and Social Security Bureau or organizations of social protection should pay attention to the development of such trainings.

Several strengths were presented in this study. Firstly, a wide coverage of 94 cities and 1 municipality in China and the large sample size endowed this study a great value to examine the relationships of Big five personality, self-efficacy and depressive symptoms among Chinese unemployed individuals. Secondly, face-to-face interviews provided a high response rate of this study and guaranteed the accuracy of the information. However, due to the cross-sectional design of this study, all findings obtained in the present study need to be confirmed in future prospective studies. And we only focused on the associations of Big five personality and self-efficacy with depressive symptoms, other protective mechanisms in addition to those studied here that are important to consider in the experience of depressive symptoms were not included.

## Conclusions

There was a high prevalence of depressive symptoms among Chinese unemployed population. After adjusting for demographic characteristics, extraversion, agreeableness and conscientiousness were all negatively associated with depressive symptoms whereas neuroticism was positively associated with depressive symptoms. In the present study, self-efficacy was found to partially mediate the relationship between extraversion/agreeableness/conscientiousness/neuroticism and depressive symptoms. Unemployed individuals who are highly extraverted/agreeable/conscientious may have high self-efficacy, which in turn would lead to lower levels of depressive symptoms. And unemployed individuals who had higher scores on neuroticism may have low self-efficacy, which in turn would lead to higher levels of depressive symptoms. These findings suggested that interventions that focus on both individuals’ personality and self-efficacy may be most successful to reduce depressive symptoms of unemployed individuals.

## Competing interests

The authors declare that they have no competing interest.

## Authors’ contributions

YW was involved in all aspects of the paper including design of the study, questionnaire survey, analysis and interpretation of data, drafting and revising the manuscript and approval of the final version. LTY, LL, XSY and JNW were all involved in questionnaire survey and draft of the manuscript. HW and LW made substantive intellectual contributions to the interpretation of data and draft of the manuscript. All authors have read and approved the final manuscript.

## Pre-publication history

The pre-publication history for this paper can be accessed here:

http://www.biomedcentral.com/1471-244X/14/61/prepub
